# Spatiotemporal Dynamics of Wetland in Dongting Lake Based on Multi-Source Satellite Observation Data during Last Two Decades

**DOI:** 10.3390/ijerph192114180

**Published:** 2022-10-30

**Authors:** Liwei Xing, Liang Chi, Shuqing Han, Jianzhai Wu, Jing Zhang, Cuicui Jiao, Xiangyang Zhou

**Affiliations:** 1 Key Laboratory of Agricultural Blockchain Application, Ministry of Agriculture and Rural Affairs/Agricultural Information Institute, Chinese Academy of Agricultural Sciences, Beijing 100081, China; 2 College of Economics, Sichuan University of Science & Engineering, Zigong 643000, China

**Keywords:** wetland, remote sensing, dynamic, random forest, J_Bh_ extension

## Abstract

Monitoring the dynamics of wetland resources has practical value for wetland protection, restoration and sustainable utilization. Dongting Lake wetland reserves are well known for both their intra-annual and inter-annual dynamic changes due to the effects of natural or human factors. However, most wetland monitoring research has failed to consider the seasonal wetlands, which is the most fragile wetland type, requiring more attention. In this study, we used multi-source time series remote sensing data to monitor three Dongting Lake wetland reserves between 2000 and 2020, and the seasonal wetlands were separated from permanent wetlands. Multispectral and indices time series were generated at 30 m resolution using a two-month composition strategy; the optimal features were then selected using the extension of the Jeffries–Matusita distance (J_Bh_) and random forest (RF) importance score; yearly wetland maps were identified using the optimal features and the RF classifier. Results showed that (1) the yearly wetland maps had good accuracy, and the overall accuracy and kappa coefficients of all wetland maps from 2000 to 2020 were above 89.6% and 0.86, respectively. Optimal features selected by J_Bh_ can improve both computational efficiency and classification accuracy. (2) The acreage of seasonal wetlands varies greatly among multiple years due to inter-annual differences in precipitation and evaporation. (3) Although the total wetland area of the three Dongting Lake wetland reserves remained relatively stable between 2000 and 2020, the acreage of the natural wetland types still decreased by 197.0 km^2^, and the change from natural wetland to human-made wetland (paddy field) contributed the most to this decrease. From the perspective of the ecological community, the human-made wetland has lower ecological function value than natural wetlands, so the balance between economic development and ecological protection in the three Dongting Lake wetland reserves requires further evaluation. The outcomes of this study could improve the understanding of the trends and driving mechanisms of wetland dynamics, which has important scientific significance and application value for the protection and restoration of Dongting Lake wetland reserves.

## 1. Introduction

Land cover and land use dynamics are some of the most important international research topics in Earth systems, with profound implications for natural ecosystems and human society [1,2]. Wetlands are vulnerable and sensitive to climate change, rapid population increase and fast economic development, and have been the major hotspots of monitoring in terms of land cover and land use change [3,4]. Studying the spatial distribution characteristics of wetland types and the complexity of their spatial–temporal dynamics can help us to understand the mutual influence between wetland ecosystems and natural or human factors, including global warming, environmental pollution and urban sprawl, which is conducive to the better protection and management of wetlands [5,6].

Monitoring long-term wetland dynamics by field investigations may be challenging due to poor site access and their labor-intensive and time-consuming nature [7,8,9]. Remote sensing images have been widely used to monitor large-scale wetland changes, with the advantage of obtaining ground information promptly and efficiently [10,11,12]. A series of global land cover products have mapped wetland distributions using remote sensing data, such as GlobalLand30 [13], FROM-GLC BUMODIS [14], GLCNMO2013 [15], DISCover [16] and GLC2000 [17]. However, these products do not include seasonal wetlands and cannot describe the inner-annual wetland changes.

Time series remote sensing data have shown an advantage in monitoring seasonal changes in vegetation and water [18,19,20]. The Advanced Very High-Resolution Radiometer (AVHRR) and Moderate-resolution Imaging Spectroradiometer (MODIS), with high temporal resolution, are always used to monitor the inner-annual dynamics of water [21,22,23,24], but the coarse spatial resolution causes misclassification and they cannot identify smaller wetlands precisely. Meanwhile, Landsat and Sentinel-2 data have a better temporal resolution, and previous research has demonstrated the potential of Landsat or Sentinel data for distinguishing wetland types [25,26,27,28]. However, there are only a few studies in which Landsat data are merged with Sentinel data to obtain an equal interval time series at 30 m spatial resolution for wetland monitoring.

Dongting Lake, a typical river-connecting lake, interacts with the Yangtze River [29]. In recent years, Dongting Lake wetlands have been changed and destroyed due to human activities and the dynamics of the hydrological regime, especially the operation of the Three Gorges Dam (TGD) [30]. There are a large number of studies monitoring wetland changes in Dongting Lake with remote sensing data [29,31]. However, most of the research is focused on flood area and water body changes, and any long-term dynamic study involving Dongting Lake wetland monitoring is usually based on a single date to identify wetlands and ignores the seasonal changes in wetlands [10,32], which leads to a lack of comparison among wetland classification results over multiple years. Dongting Lake, with smooth relief, tends to be influenced by seasonal precipitation, and may undergo significant variation among different seasons [33]. There is an urgent requirement to monitor the wetlands’ intra-annual and inter-annual dynamics in Dongting Lake using satellite series time at a high spatial resolution. Therefore, the main objectives of this study are (1) to generate yearly wetland maps in the three Dongting Lake wetland reserves at 30 m spatial resolution, and (2) to analyze the seasonal and inter-annual dynamics of wetlands in Dongting Lake wetland reserves from 2000 to 2020 and reveal the motivation and regulation of these dynamics.

## 2. Study Area and Materials

### 2.1. Study Area

**Figure 1:**
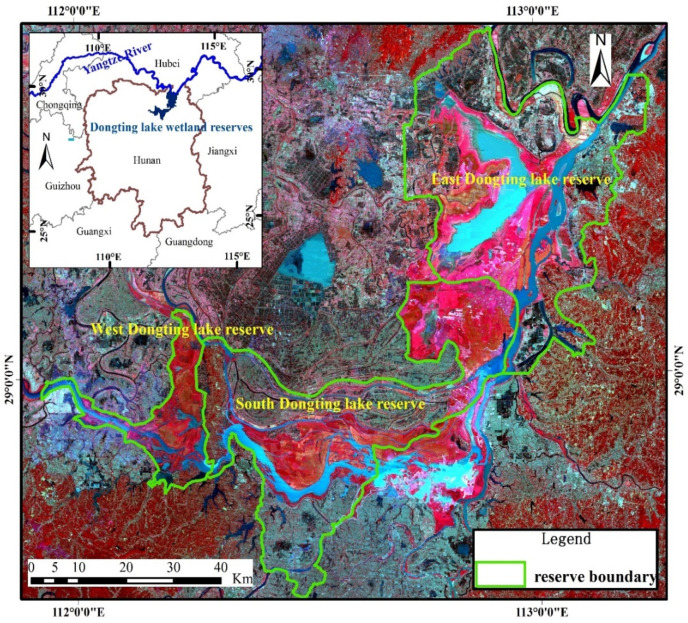
Location of study area.

Dongting Lake is located in the north of Hunan Province (28°30′~30°20′ N, 110°40′~113°10′ E), and is the second-largest freshwater lake in China [29]. The lake has a subtropical monsoon climate that is characterized by a rainy season between April and September and a dry season between October and March. Dongting Lake, with smooth relief, tends to be influenced by seasonal precipitation, and may undergo significant changes over several days [34]. During the rainy season, the lake provides storage for river flood waters. During the dry season, it provides water to the river to allow river transportation to continue without significant interruption. Seasonal mudflats provide rare habitats and spawning grounds for migratory birds and fish [35]. High-frequency measurements are, therefore, needed to study short- and long-term fluctuations in the inundation areas of lakes. Dongting Lake includes three national wetland nature reserves (Figure 1), the East Dongting Lake wetland reserve, the South Dongting Lake wetland reserve and the West Dongting Lake wetland reserve, which were included in the list of Ramsar Sites in 1992 and 2002 [7]. Dongting Lake wetland reserves include various types of land cover. In this study, the classification system uses a two-tier hierarchical structure. Level 1 comprises two categories: wetlands and uplands. Level 2 comprises 7 categories: four types of natural wetlands (permanent water, permanent marsh, flooded wetland and seasonal marsh), an artificial wetland (paddy field), one type of natural upland (forest) and one type of artificial upland (construction land).

### 2.2. Materials

#### 2.2.1. Remote Sensing Data

Remote sensing data with higher temporal resolution are of key importance for mapping seasonal wetlands. A series of medium-resolution satellites at 20–30 m spatial resolution enable us to map and monitor seasonal wetlands. In this study, all Landsat-5, Landsat-7, Landsat-8 and Sentinel-2 data between 2000 and 2020 (Table 1) were prepared and preprocessed on Google Earth Engine [36,37]. In addition, the top of atmosphere (TOA) reflectance of both Landsat 5/7/8 and Sentinel-2 was utilized because surface reflectance (SR) data were not available on GEE before 2019; this is appropriate as existing studies have shown that the correlations of Landsat and Sentinel-2 for TOA reflectance data are higher than those for SR data [38,39]. Finally, 3235 images were used in total, including 551 Landsat-5 TM images, 987 Landsat-7 ETM+ images, 425 Landsat-8 OLI images and 1272 Sentinel-2 MSI images (Table 1). All data were re-sampled to 30 m, and quality assurance (QA) bands were used to mask cloud pixels. Afterwards, two-month image time series were generated using the two-month median composition for the multi-spectral bands. We used a two-month composition strategy in this study because it is the best choice to compensate for the missing data and retain the seasonal variation in the land surface. Finally, we acquired the normalized difference water index (NDWI) [40], normalized difference moisture index (NDMI) [41] and normal difference vegetation index (NDVI) [42] time series from the two-month composited images. These indices were selected because NDWI time series have been successfully used for detecting permanent and seasonal water (Equation (1)); NDMI time series have been proven to increase the separability among wetland plant varieties (Equation (2)), and they can determine the phenological parameters of wetland plants (Equation (3)).

(1) $$\mathrm{NDWI}=\frac{\rho \left(\mathrm{Green}\right)-\rho \left(\mathrm{NIR}\right)}{\rho \left(\mathrm{Green}\right)+\rho \left(\mathrm{NIR}\right)}$$

(2) $$\mathrm{NDMI}=\frac{\rho \left(\mathrm{NIR}\right)-\rho \left(\mathrm{SWIR}1\right)}{\rho \left(\mathrm{NIR}\right)+\rho \left(\mathrm{SWIR}1\right)}$$

(3) $$\mathrm{NDVI}=\frac{\rho \left(\mathrm{NIR}\right)-\rho \left(\mathrm{Red}\right)}{\rho \left(\mathrm{NIR}\right)+\rho \left(\mathrm{Red}\right)}$$

where ρ(Green), ρ(Red), ρ(NIR) and ρ(SWIR1) denote the TOA reflectance of the Green, Red, NIR and SWIR1 bands, respectively, which are Band2, Band3, Band4 and Band5 for Landsat-5 and Landsat-7 data; Band3, Band4, Band5 and Band6 for Landsat-8; and Band3, Band4, Band8 and Band11 for Sentinel-2 (Table 2).

#### 2.2.2. Training and Validation Samples

**Figure 2:**
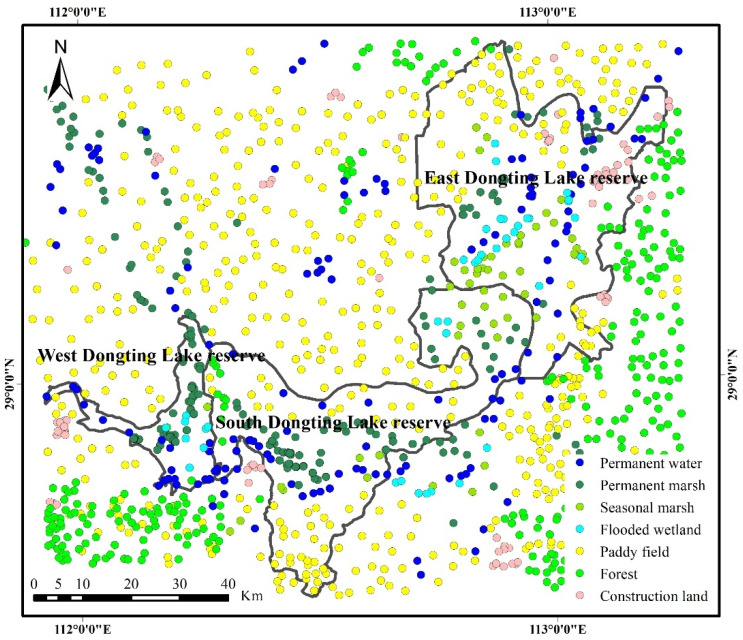
Distribution of samples for each land cover type in 2020.

In this study, training and validation samples were used to train the classifier and evaluate the accuracy of the classification results, respectively. Initially, 2000 geolocation sampling points were randomly generated in the study region using ArcGIS (ESRI, Redlands, SC, USA) for each year. Subsequently, the samples were visually interpreted as permanent water, permanent marsh, flooded wetland, seasonal marsh, paddy field, forest and construction land based on remote sensing images with high spatial resolution from Google Earth (http://earth.google.com, accessed on 5 January 2022) and the characteristics of the reconstructed two-month composition time series data set (NDVI, NDWI and NDMI) for each land cover in the same year. Only the samples with 120 m × 120 m pure pixels were retained (Figure 2). Finally, these samples in each year were divided into two parts for training and validation; two thirds of these samples were randomly selected and used as training samples to classify, and the remaining one third of the samples were used as validation samples to verify the accuracy of the classification results.

## 3. Methodology

### 3.1. Flowchart

**Figure 3:**
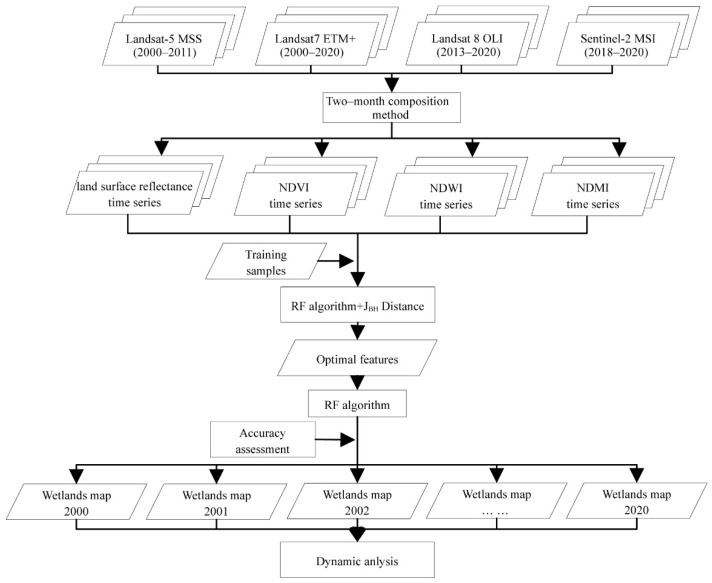
Flowchart of the methodology followed in this study.

The framework for mapping seasonal wetlands developed in this paper is shown in Figure 3. We generated two-month time series with 54 features, including multi-spectral bands and indices (NDWI, NDMI and NDVI) for all time phases. To select the optimal features and improve the classification efficiency, importance scores for all these features were calculated based on the random forest algorithm using the training samples, and the ranking of importance scores for all 54 features were acquired. Afterwards, we calculated the separability among all wetland types using the extension of the Jeffries–Matusita (JM) distance (J_Bh_); the number of features used increased from 1 to 54, and the sequence of adding features was based on the descending order of the importance score. In other words, the features with a higher importance score were added to the feature set, which was used for separability calculation, in higher priority. The separability among all wetland types increased with the increase in the number of features, and it was saturated when the features reached a certain amount; the features before the saturation points were then used as optimal features. Next, the RF algorithm was used to classify the wetland types based on the optimal features, and the classification accuracy was calculated to measure the classification performance. Finally, we analyzed the dynamics of wetland areas in the three Dongting Lake wetland reserves from 2000 to 2020 based on the wetland maps that we generated.

### 3.2. Random Forest

The most commonly used classification algorithms for wetlands are maximum likelihood (ML, [43]), decision tree (DT, [44]), random forest (RF, [44]), and support vector machine (SVM, [45]). In our study, the RF algorithm was chosen for wetland mapping. It has high efficiency and flexibility and is suitable for large datasets. RF is an ensemble-based machine learning algorithm that combines a set of Classification and Regression Trees (CARTs). Two thirds of the samples were used to train each tree. The remaining one third of samples was then used to validate the classification result, with an error called the “out-of-bag (OOB) error”. Next, the final output was determined by majority voting on all classification results obtained by each tree. Two parameters needed to be set in the RF algorithm: the number of decision trees to be generated (Ntree) and the number of features to best split each node (Mtry). Another function of RF is to derive the importance of each feature. In this study, the RF package for R was used to calculate the importance scores of features and classify wetland types. The Ntree was set to 1000 to ensure that the OOB errors stabilized and reached convergence. The Mtry was set to the square root of the number of input features.

### 3.3. J_Bh_ Extention Method

The Jeffries–Matusita (JM) distance was selected to estimate the separability of wetland types, as many studies showed that the JM distance can measure the separability more accurately than other distance measures [46]. The JM distance between two classes was given by Equation (4):

(4) $$\mathrm{JM}\left(c_{i},c_{j}\right)=\int_{x}^{}{\left[\sqrt{p\left(X/c_{i}\right)}-\sqrt{p\left(X/c_{j}\right)}\right]}^{2}\;dX$$

where *c_i_* and *c_j_* represented the two wetland classes. Under normality assumptions, Equation (4) was reduced to JM = 2·(1 − e^−B^), where

(5) $$B=\frac{1}{8}\left(u_{i}-u_{j}\right){\left(\frac{C_{i}+C_{j}}{2}\right)}^{T}\left(u_{i}-u_{j}\right)+\frac{1}{2}\ln\left(\left|\frac{\left|C_{i}+C_{j}\right|}{2\sqrt{\left|C_{i}\right|*\left|C_{j}\right|}}\right|\right)$$

and *C_i_* and *C_j_* represented the covariance matrices of classes *i* and *j*, respectively. $\left|C_{i}\right|$ and $\left|C_{j}\right|$ denoted the determinants of *C_i_* and *C_j_*, respectively. The range of JM distance was from 0 to 2. A high value indicated high separability between the two classes [47].

When considering the separability of multiple classes, the extension of the JM distance (J_Bh_) was used in this study. The J_Bh_ can be calculated as in Equation (6) based on Bhattacharyya bounds [48].

(6) $$J_{\mathrm{Bh}\;=}\sum_{i=1}^{N}\sum_{j>i}^{N}\sqrt{p\left(w_{i}\right)*p\left(w_{j}\right)}*JM^{2}\left(i,j\right)\;$$

where *N* was the number of classes, and *p*(*w_i_*) and *p*(*w_j_*) were the prior probability of class *i* and class *j* calculated using training samples.

### 3.4. Accuracy Evaluation

In this study, the accuracy of the wetland classification results was evaluated as the overall accuracy (OA) (Equation (7)), user’s accuracy (UA) (Equation (8)), producer’s accuracy (PA) (Equation (9)) and kappa coefficient (Equation (10)). These parameters can be calculated based on the confusion matrix [49].

(7) $$p_{o}=\sum_{i=1}^{r}p_{ii}/N$$

(8) $$p_{u_{i}}=p_{ii}/p_{i+}$$

(9) $$p_{A_{i}}=p_{ii}/p_{+i}$$

(10) $$Kappa=\frac{N*\sum_{i=1}^{r}p_{ii}-\sum_{i=1}^{r}\left(p_{i+}*P_{+i}\right)}{N^{2}-\sum_{i=1}^{r}\left(p_{i+}*p_{+i}\right)}$$

where $p_{o}$ is the overall accuracy; $p_{u_{i}}$ is the user’s accuracy; $p_{A_{i}}$ is the producer’s accuracy; $p_{ii}$ is the number of correctly classified samples of class *i*; *r* is the number of classes; *N* is the number of training samples; $p_{i+}$ is the number of classified samples of class *i*; and $p_{+i}$ is the number of training samples of class *i*.

## 4. Results and Discussion

### 4.1. Classification Accuracy

**Figure 4:**
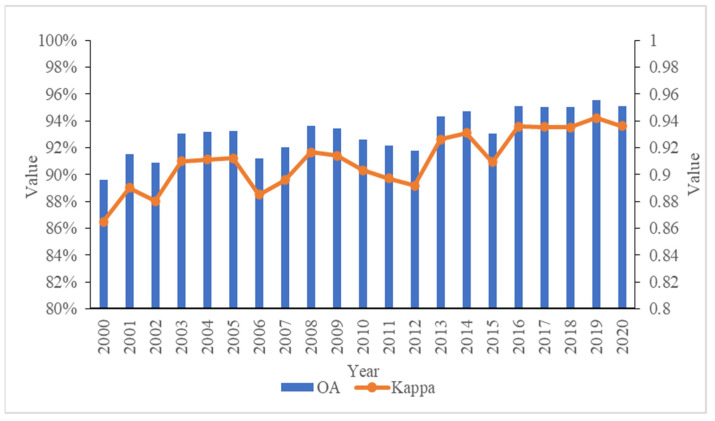
Overall accuracy (OA) and kappa coefficient for each year.

Validation samples were used to verify the wetland classification results generated in this study. OA, PA, UA and the kappa coefficient calculated from the confusion matrix were used to evaluate the wetland classification accuracy. The results showed that the wetland maps in the study had high accuracy. The OA ranged from 89.6% to 95.6%, with an average value of 93.1%. The kappa coefficient ranged from 0.86 to 0.94, with an average value of 0.91 (Figure 4). The producer’s and user’s accuracies for each land cover type were above 72.9% and 73.9%, respectively (Table 3). Both PA and UA for permanent water, permanent marsh, paddy field and seasonal marsh were high and stable for each year, with averages of 96.7%, 95.8%, 95.3%, 91.7% and 95.3%, 95.8%, 93.9%, 94.4%, respectively. The PA and UA only for flooded wetland were below 90%, with averages of 88.3% and 89.7%, respectively. The main omissions or commissions of flooded wetland were attributed to its smaller area, with the problem of mixed pixels. In general, the classification results in our study are reliable and acceptable for monitoring wetland changes in Dongting Lake wetland reserves.

### 4.2. Wetland Types and Their Distributions

**Figure 5:**
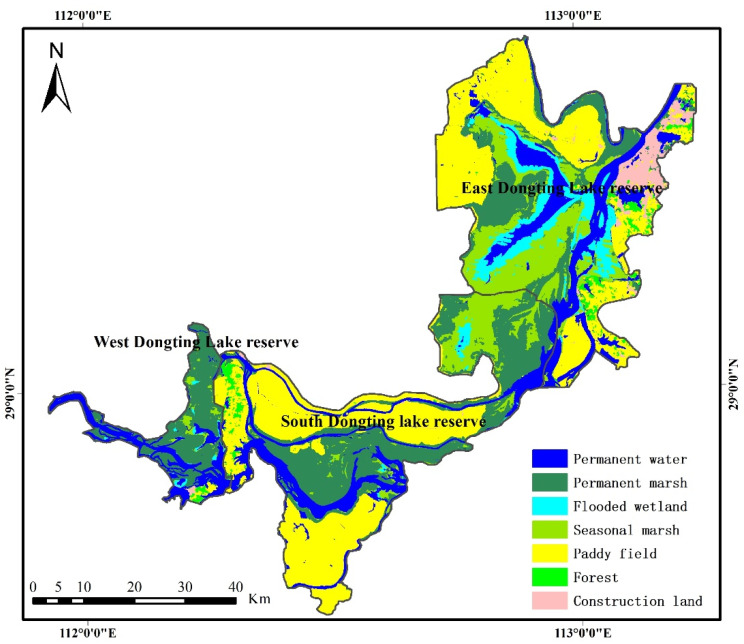
The classification result of the study area in 2020.

**Figure 6:**
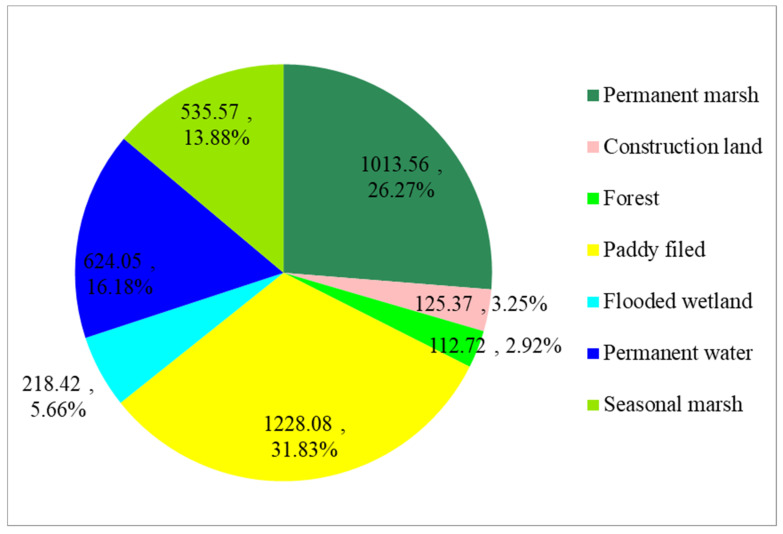
Area ratio of each land cover type in 2020.

The inter-annual Dongting Lake wetland classification results were obtained using optimal features and the RF algorithm. The results showed that the total area of wetlands in Dongting Lake was 3619.67 km^2^ in 2020 (Figure 5), accounting for more than 93% of the total area of the three wetland reserves. Natural wetlands, including permanent water (624.05 km^2^), permanent marsh (1013.56 km^2^), seasonal marsh (535.57 km^2^) and flooded wetlands (218.42 km^2^), accounted for 61.99% of the total area of the three wetland reserves. The paddy field area in 2020 was 1228.08 km^2^, accounting for 31.83% of the total area of the three wetland reserves (Figure 6).

**Figure 7:**
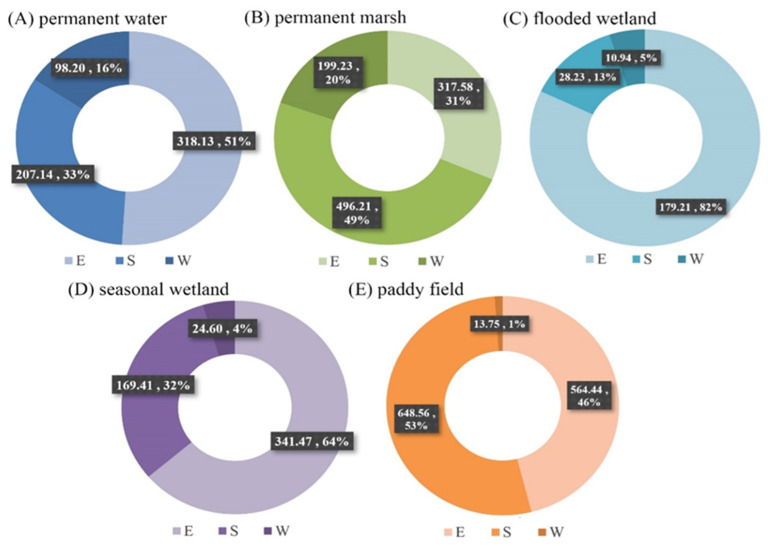
Spatial characteristics of different wetlands in the three Dongting Lake wetland reserves. Note: E represents the East Dongting Lake wetland reserve; S represents the South Dongting Lake wetland reserve; W represents the West Dongting Lake wetland reserve.

Figure 7 shows the spatial distribution of wetland types in these three reserves. Almost all natural wetlands (Figure 7A–D) and human-made wetlands (Figure 7E) were located in the East Dongting Lake wetland reserve and South Dongting Lake wetland reserve, accounting for 99% and 86% of their respective total areas. More than 50% of permanent water (Figure 7A), with an area of 318.13 km^2^, was located in the East Dongting Lake wetland reserve, and 82% of flooded wetland (Figure 7C) was also located in the East Dongting Lake wetland reserve, as the central district of Dongting Lake is located in the East Dongting Lake wetland reserve and the flooded wetland was generally distributed around permanent water. Permanent marsh (Figure 7B) was mainly located in the South Dongting Lake wetland reserve, accounting for 49% of the total area of permanent marsh, while seasonal marsh (Figure 7D) was mainly located in the East Dongting Lake wetland reserve.

**Figure 8:**
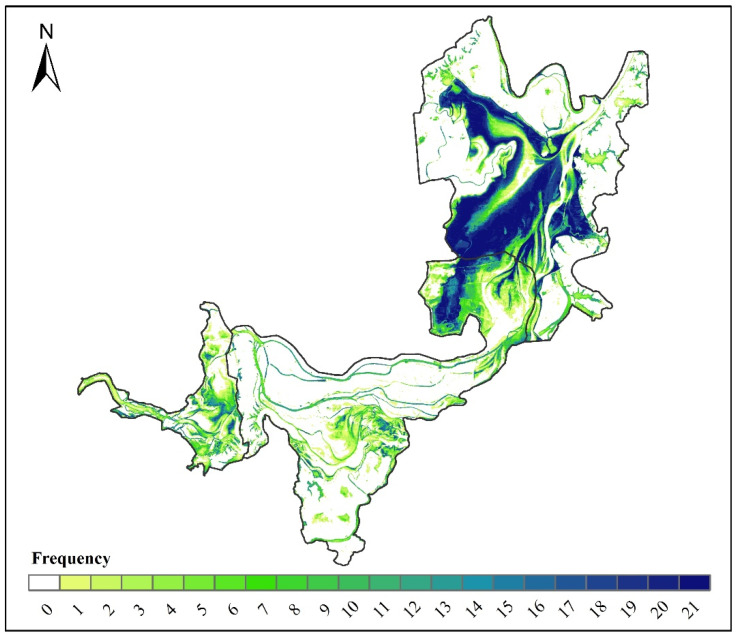
Spatiotemporal dynamic changes in seasonal wetlands. Note: The numbering 0–21 reflects the number of seasonal wetland occurrences in the same location from 2000 to 2020.

Seasonal wetlands, with high heterogeneity of temporal and spatial changes, have more abundant ecological functions and higher biodiversity than permanent wetlands [50,51]. Figure 8 shows the spatiotemporal dynamic changes in the seasonal wetlands at the three Dongting Lake wetland reserves, by overlaying the mapping results of the seasonal wetlands from 2000 to 2020. The seasonal wetlands were always distributed between permanent water and permanent marsh. The overall inter-annual change characteristics are that seasonal wetlands occur less frequently near permanent water and permanent marsh. However, the occurrence frequency of seasonal wetlands increases with the increase in distance, and decreases after a certain distance. In the East Dongting Lake wetland reserve, the spatial distribution of seasonal wetlands remained stable during 2000 and 2020 (frequency = 21); in the South Dongting Lake wetland and West Dongting Lake wetland reserves, most pixels that were identified as seasonal wetlands were classified as other land cover types in other years. The total area of land that was covered by seasonal wetlands (0 < frequency ≤ 21) during the 21 years was 2079.3 km^2^, accounting for more than 53.9% of the total area of the three Dongting Lake wetland reserves, while the acreage of the land dominated by seasonal wetlands in all 21 years (frequency = 21) was 252.9 km^2^, accounting for only 6.6% of the total area of the three Dongting Lake wetland reserves. Therefore, seasonal wetlands are seriously affected by inter-annual fluctuations, such as rainfall, temperature, and human activities, and more attention should be paid to this specific wetland type considering its ecological value and vulnerability [33]. 

**Figure 9:**
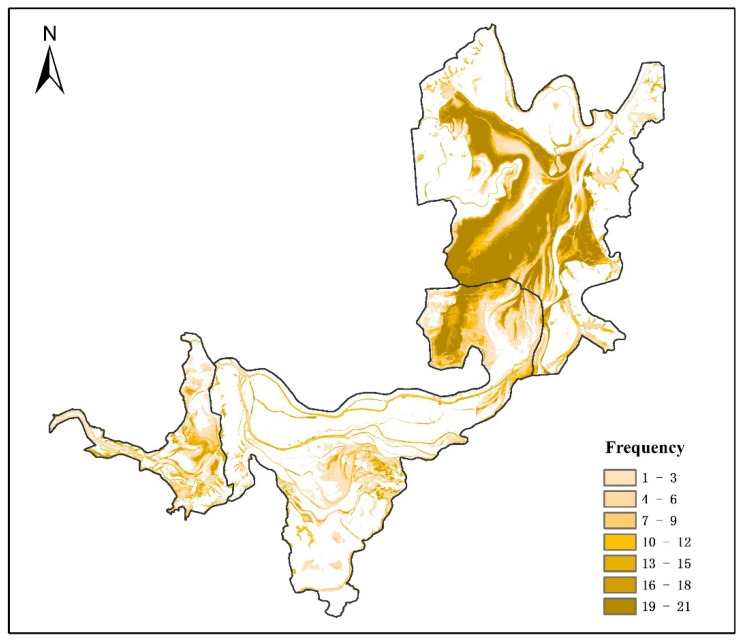
The spatial characteristics of seven levels’ frequencies.

**Figure 10:**
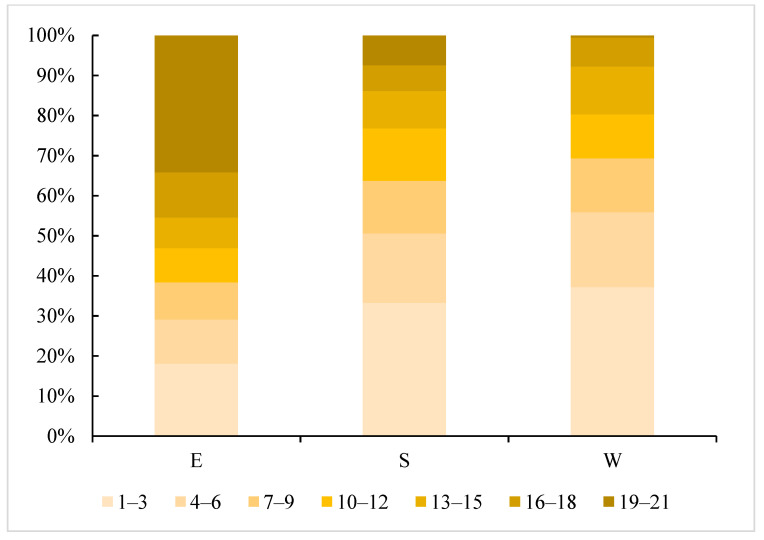
The proportion of the seven levels’ frequencies in the three wetland reserves.

As a whole, the acreage of seasonal wetlands (0 < frequency ≤ 21) in the East Dongting Lake wetland reserve, the South Dongting Lake wetland reserve and the West Dongting Lake wetland reserve was 1190.3 km^2^, 668.7 km^2^ and 220.2 km^2^, accounting for 62.8%, 41.7% and 61.8% of each wetland reserve, respectively. Meanwhile, there were still some differences in the spatial characteristics of these three wetland reserves (Figure 9). In the East Dongting wetland reserve, the high-frequency seasonal wetland region (19 ≤ frequency ≤ 21) accounted for the largest proportion of the seasonal wetlands (0 < frequency ≤ 21) area (34%). However, the low-frequency seasonal wetland region (1 ≤ frequency ≤ 3) accounted for the largest proportion in both the South Dongting Lake wetland reserve (33%) and the West Dongting Lake wetland reserve (37%) (Figure 10). Thus, the inter-annual variation in seasonal wetlands is small in the East Dongting Lake wetland reserve, but large in the South/West Dongting Lake wetland reserve.

### 4.3. Wetland Changes

**Figure 11:**
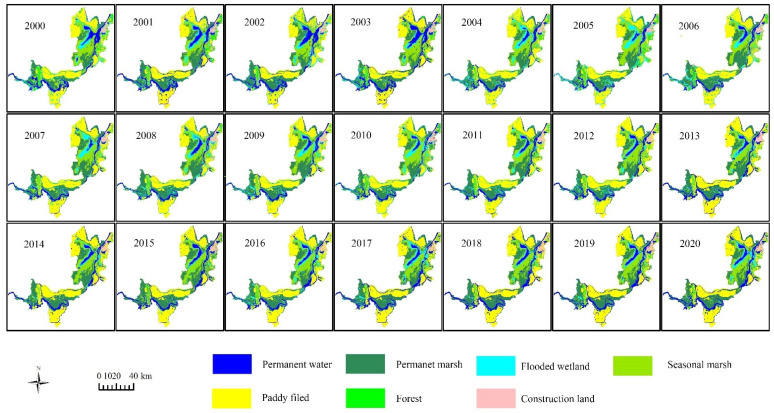
Spatiotemporal distribution of classification results of the three Dongting Lake wetland reserves from 2000 to 2020.

**Figure 12:**
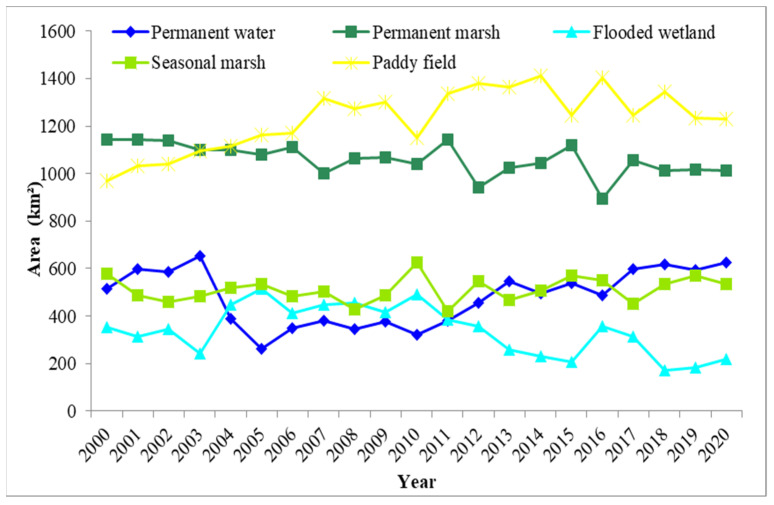
Dynamic changes in the three Dongting Lake wetland reserves from 2000 to 2020.

Figure 11 shows the yearly classification results of the three Dongting Lake wetland reserves between 2000 and 2020. The total wetland area of the three Dongting Lake wetland reserves slightly increased by 63.1 km^2^ from 2000 to 2020, accounting for 1.6% of the total area of the three reserves. This means that the wetland area of these three reserves remained stable. However, the acreage of natural wetland (permanent water, permanent marsh, flooded wetland and seasonal marsh) decreased year by year, due to the area decline of permanent marsh, flooded wetland and seasonal marsh. The area of natural wetlands decreased by 197.0 km^2^ from 2000 to 2020 (Figure 12). The natural wetland area in 2000 was 2588.6 km^2^, and it decreased to 2480.8 km^2^ in 2010. From 2010 to 2020, the natural wetland area decreased to 2391.6 km^2^. Human-made wetlands (paddy fields) increased by 260.0 km^2^ during the two decades, but the main function of human-made wetlands is to create economic value. Some researchers have found that the ecological functions of natural wetlands are far greater than the economic benefits [52], and the expansion of paddy fields also places pressure on water resources. Therefore, it is crucial to balance the agriculture development and ecology functions and implement some management measures to protect the natural wetlands in the three Dongting Lake wetland reserves.

### 4.4. Influencing Factor Analysis

**Figure 13:**
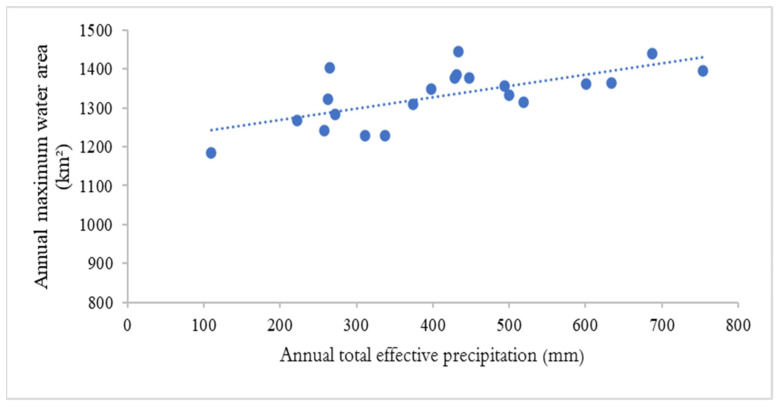
Linear regression analysis of the annual total effective precipitation and annual maximum water area of the three Dongting Lake wetland reserves from 2000 to 2020.

There were a number of factors affecting the surface water acreage of Dongting Lake, such as precipitation, evaporation, runoff, and infiltration. Monthly precipitation and evaporation data from 2000 to 2020 were acquired from the GLDAS Noah Land Surface Model L4 monthly 0.25 × 0.25 degree V2.1 [53], which were downloaded from the NASA Earth Data platform (https://www.earthdata.nasa.gov/, accessed on 6 April 2022). In our study, effective monthly precipitation equals the monthly precipitation minus the monthly evaporation. Then, the annual effective precipitation is obtained by summing the monthly effective precipitation. We used the annual effective precipitation to analyze the potential factors involved in the annual maximum surface water acreage variation. The annual maximum surface water area was the total acreage of seasonal marsh, flooded wetlands and permanent water bodies. Results showed a high positive correlation between the annual maximum surface water and annual effective precipitation (correlation coefficient r = 0.7, *p* < 0.001), which indicates that the annual maximum surface water area of Dongting Lake increased as the annual effective precipitation increased, and it deceased as the annual effective precipitation decreased (Figure 13). Therefore, the effective precipitation was one of the most important factors that influenced the dynamic changes in the surface water area of Dongting Lake.

## 5. Conclusions

In this study, we used all available satellite images at 10~30 m spatial resolution to generate yearly wetland maps of the three Dongting Lake wetland reserves between 2000 and 2020, and monitored the wetland dynamics throughout the two decades; the classification categories included seasonal wetlands, which have high ecological value but are often omitted in existing studies. The main conclusions are as follows:

(1) Using a two-month composition to construct time series data, we effectively eliminated the influence of clouds and strips. The reconstructed two-month composition time series data set (NDVI, NDWI and NDMI) could effectively reflect the information on wetland phenology and water inundation. The results showed that the two-month composition strategy had good potential to be used as basic data for yearly wetland distribution mapping, and this strategy effectively improves the utilization of multi-source remote sensing data.
(2) The use of optimal features and the random forest classifier achieved good wetland identification accuracies, as the OA and kappa coefficients of our classification results were above 89.6% and 0.86, respectively. The PA and UA for all land cover types were above 72.9% and 73.9%, respectively. Feature optimization not only reduces data redundancy and improves operation efficiency, but also achieves wetland identification. However, due to the different characteristics of wetland vegetation in different regions, the optimal features are different.
(3) The total area of wetlands (including natural and human-made wetlands) in these three Dongting Lake wetland reserves essentially remained stable between 2000 and 2020. Although human-made wetlands (paddy fields) increased by 260.0 km^2^, the area of natural wetlands decreased by 197.0 km^2^. The acreage of seasonal wetlands decreased by 176.8 km^2^, which was affected by both human factors (farmland expansion) and natural factors (precipitation and evaporation).

Therefore, more attention should be focused on the fragile seasonal wetlands, which play a vital role in promoting species diversity and supporting their survival. In our study, we only analyzed the correlation between the annual maximum surface water acreage and the annual effective precipitation. As more meteorological, human and economic data are obtained, the detailed reasons for the annual dynamics of these three Dongting Lake reserves will be further explored in the future.

## Figures and Tables

**Table 1:** The number of images used in our study.

Number	Landsat-5	Landsat-7	Landsat-8	Sentinel-2	SUM
2000	37	44	0	0	81
2001	52	54	0	0	106
2002	38	52	0	0	90
2003	42	37	0	0	79
2004	58	47	0	0	105
2005	49	51	0	0	100
2006	50	50	0	0	100
2007	33	48	0	0	81
2008	55	55	0	0	110
2009	58	54	0	0	112
2010	38	45	0	0	83
2011	41	49	0	0	90
2012	0	38	0	0	38
2013	0	52	58	0	110
2014	0	40	47	0	87
2015	0	45	60	0	105
2016	0	48	53	0	101
2017	0	42	51	0	93
2018	0	45	58	30	133
2019	0	43	52	623	718
2020	0	48	46	619	713
SUM	551	987	425	1272	

**Table 2:** Introduction to bands.

Generic Name	Landsat-5	Landsat-7	Landsat-8	Sentinel-2
Blue	1 (450–520)	1 (450–520)	2 (450–510)	2 (458–522)
Green	2 (520–600)	2 (520–600)	3 (530–590)	3 (543–578)
Red	3 (630–690)	3 (630–690)	4 (640–670)	4 (650–680)
Near-Infra-Red (NIR)	4 (760–900)	4 (770–900)	5 (850–880)	8 (785–900)
Short-Wave Infra-Red 1 (SWIR1)	5 (1550–1750)	5 (1550–1750)	6 (1570–1650)	11 (1565–1655)
Short-Wave Infra-Red 2 (SWIR2)	7 (2080–2350)	7 (2090–2350)	7 (2110–2290)	12 (2100–2280)

**Table 3:** Classification accuracy for each year.

Year	Permanent Water	Permanent Marsh	Flooded Wetland	Seasonal Marsh	Paddy Field	Forest	Construction Land
	PA/UA	PA/UA	PA/UA	PA/UA	PA/UA	PA/UA	PA/UA
2000	97.44%/95%	95.02%/94.17%	89.13%/93.18%	96.77%/96.77%	92.4%/91.07%	79.07%/81.93%	72.86%/73.91%
2001	94.85%/97.87%	93.67%/92.83%	82.14%/82.14%	94.44%/91.89%	93.57%/93.02%	88.95%/88.44%	78.57%/82.09%
2002	96.67%/95.6%	96.24%/95.88%	81.25%/83.87%	82.35%/93.33%	95.32%/87.87%	77.91%/85.9%	81.43%/95%
2003	97.06%/97.06%	92.48%/96.09%	84.62%/81.48%	83.33%/94.59%	95.61%/95.61%	95.35%/82.41%	80%/98.25%
2004	96.34%/95.18%	98.12%/96.67%	81.25%/86.67%	81.08%/93.75%	94.74%/92.84%	91.86%/90.8%	78.57%/87.3%
2005	90.91%/88.24%	95.49%/96.58%	85.96%/89.09%	93.33%/90.32%	94.15%/95.55%	93.02%/86.49%	88.57%/96.88%
2006	98.63%/97.3%	92.48%/90.44%	94.29%/97.06%	96.97%/94.12%	93.86%/92.51%	80.23%/84.66%	88.57%/92.54%
2007	97.3%/96%	99.25%/96.7%	86.84%/94.29%	90.63%/93.55%	93.57%/88.89%	79.65%/88.39%	85.71%/92.31%
2008	97.14%/93.15%	95.11%/93.7%	80.56%/93.55%	93.94%/91.18%	93.57%/92.22%	94.77%/95.88%	88.57%/96.88%
2009	95.83%/98.57%	95.49%/95.49%	96.97%/88.89%	97.06%/94.29%	95.03%/93.12%	87.79%/92.07%	85.71%/86.96%
2010	93.55%/87.88%	99.25%/94.29%	82.69%/91.49%	90.63%/93.55%	93.57%/96.1%	88.37%/89.41%	80%/81.16%
2011	97.22%/97.22%	94.36%/94.36%	91.43%/94.12%	92.59%/96.15%	93.86%/91.98%	87.21%/89.29%	82.86%/84.06%
2012	97.22%/89.74%	96.99%/95.56%	82.05%/88.89%	87.5%/96.55%	95.32%/91.57%	81.4%/88.05%	81.43%/87.69%
2013	97.5%/95.12%	95.49%/97.69%	88.24%/88.24%	94.87%/97.37%	97.37%/95.42%	90.7%/91.23%	82.86%/84.06%
2014	97.33%/97.33%	96.62%/99.23%	93.33%/87.5%	95.35%/95.35%	97.95%/95.71%	89.53%/91.67%	81.43%/80.28%
2015	98.67%/96.1%	95.49%/94.42%	85.29%/85.29%	88.24%/93.75%	95.32%/94.49%	88.95%/93.29%	82.86%/80.56%
2016	98.67%/97.37%	98.5%/98.5%	94.12%/91.43%	94.12%/96.97%	96.78%/96.78%	90.12%/91.18%	82.86%/81.69%
2017	96%/96%	96.62%/98.47%	92.86%/88.64%	94.44%/94.44%	97.95%/97.1%	91.28%/91.81%	84.29%/94.12%
2018	97.22%/97.22%	93.61%/96.14%	97.14%/91.89%	97.67%/97.67%	95.91%/96.19%	94.77%/90.56%	91.43%/94.12%
2019	97.18%/95.83%	96.99%/98.85%	94.12%/91.43%	94.74%/97.3%	98.25%/97.67%	91.81%/91.28%	85.71%/84.51%
2020	98.84%/97.7%	95.13%/96.58%	91.3%/95.45%	86.54%/90%	97.95%/96.82%	93.02%/92.49%	88.57%/87.32%

Note: UA, user accuracy; PA, producer accuracy.

## Data Availability

Not applicable.
